# Dibromido(2,2′:6′,2′′-terpyridine-κ^3^ *N*,*N*′,*N*′′)zinc(II)

**DOI:** 10.1107/S1600536809019266

**Published:** 2009-05-29

**Authors:** Qing-Lan Zhao, Guo-Peng Li

**Affiliations:** a College of Chemistry and Chemical Engineering, Henan University, Kaifeng 475001, Henan, People’s Republic of China; b Institute of Molecular and Crystal Engineering, College of Chemistry and Chemical Engineering, Henan University, Kaifeng 475001, Henan, People’s Republic of China

## Abstract

In the title compound, [ZnBr_2_(C_15_H_11_N_3_)], the Zn^II^ ion is five-coordinated by the three N atoms from a 2,2′:6′,2′′-terpyridine ligand (terpy) and two bromide anions in a distorted trigonal bipyramidal configuration. Each mol­ecule is situated on a twofold rotational axis that passes through the Zn^II^ ion and the central ring of the terpy ligand. In the crystal structure, aromatic π–π inter­actions between terpy ligands [centroid–centroid distances = 3.6265 (9) Å] link mol­ecules into stacks propagated in the [001] direction.

## Related literature

For related structures, see: Alizadeh *et al.* (2009 ▶); Mahmoudi *et al.* (2009 ▶); Huang *et al.* (2009 ▶); Ma *et al.* (2009 ▶); Bai *et al.* (2009 ▶).
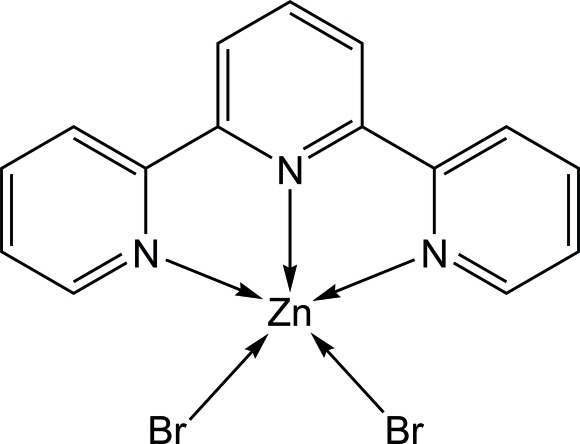


## Experimental

### 

#### Crystal data

[ZnBr_2_(C_15_H_11_N_3_)]
*M* *_r_* = 458.46
Monoclinic, 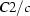

*a* = 17.0972 (5) Å
*b* = 9.3528 (3) Å
*c* = 11.5334 (4) Å
β = 126.051 (1)°
*V* = 1491.08 (8) Å^3^
*Z* = 4
Mo *K*α radiation
μ = 7.00 mm^−1^
*T* = 296 K
0.20 × 0.18 × 0.16 mm

#### Data collection

Bruker SMART APEXII CCD diffractometer
Absorption correction: multi-scan (*SADABS*; Bruker, 2005 ▶) *T*_min_ = 0.335, *T*_max_ = 0.401 (expected range = 0.273–0.326)
9665 measured reflections
1457 independent reflections
1371 reflections with *I* > 2σ(*I*)
*R*_int_ = 0.019

#### Refinement

*R*[*F* ^2^ > 2σ(*F* ^2^)] = 0.015
*wR*(*F* ^2^) = 0.039
*S* = 1.08
1457 reflections
97 parameters
H-atom parameters constrained
Δρ_max_ = 0.27 e Å^−3^
Δρ_min_ = −0.29 e Å^−3^

### 

Data collection: *APEX2* (Bruker, 2005 ▶); cell refinement: *SAINT* (Bruker, 2005 ▶); data reduction: *SAINT*; program(s) used to solve structure: *SHELXS97* (Sheldrick, 2008 ▶); program(s) used to refine structure: *SHELXL97* (Sheldrick, 2008 ▶); molecular graphics: *SHELXTL* (Sheldrick, 2008 ▶); software used to prepare material for publication: *SHELXTL*.

## Supplementary Material

Crystal structure: contains datablocks I, global. DOI: 10.1107/S1600536809019266/cv2561sup1.cif

Structure factors: contains datablocks I. DOI: 10.1107/S1600536809019266/cv2561Isup2.hkl

Additional supplementary materials:  crystallographic information; 3D view; checkCIF report

## Figures and Tables

**Fig. 1.**
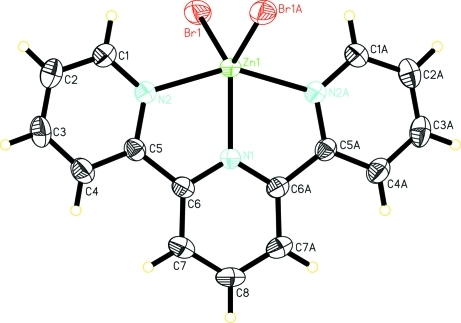
The molecular structure of the title compound with the atom-labelling scheme [symmetry code: (A) -x, + 1, y, -z + 1/2]. Displacement ellipsoids are drawn at the 50% probability level. H atoms are presented as small spheres of arbitrary radius.

**Fig. 2.**
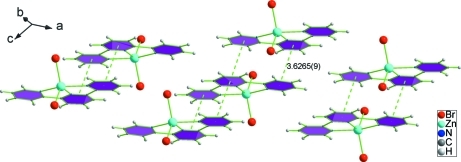
A portion of the crystal packing showing the π–π interactions (dashed lines) between the aromatic rings.

## supplementary crystallographic information

### Comment

As a contribution to structural characterization of 2,2':6',2''-terpyridine complexes (Alizadeh *et al.*, 2009; Huang *et al.*, 2009; Ma *et al.*, 2009; Bai *et al.*, 2009) we present here the title complex (I).

In (I) (Fig. 1), the Zn^II^ ion is five-coordinated in a distorted trigonal bipyramidal configuration by three N atoms from a 2,2':6',2''-terpyridine ligand and by two Br anions. The Zn–Br and Zn–N bond lengths are within normal ranges (Mahmoudi *et al.*, 2009).

In the crystal structure, the π–π stacking interactions between aromatic rings of *Cg*1 and *Cg*2 [*Cg*1 and *Cg*2 are (N1, C6 — C8, C7^i^, C6^i^) and (N2, C1 — C5) ring centroids, respectively, symmetry code: (i) -*x* + 1, *y*, -*z* + 1/2] are observed, with a centroid–centroid distances of 3.6265 (9) Å.

### Experimental

The title compound was synthesized hydrothermally in a Teflon-lined autoclave (25 mL) by heating a mixture of 2,2':6',2''-terpyridine (0.2 mmol), ZnBr_2_ (0.2 mmol) and one drop of Et_3_N (pH ≈ 8–9) in water (10 mL) at 393 K for 3 d. Crystals suitable for X-ray analysis were obtained.

### Refinement

All H atoms were included in calculated positions, with C—H distances fixed to 0.93 Å and were refined in the riding-model approximation, with *U*_iso_(H) = 1.2 *U*_eq_ (C).

### Crystal data

**Table:**

[ZnBr_2_(C_15_H_11_N_3_)]	*F*(000) = 888
*M**_r_* = 458.46	*D*_x_ = 2.042 Mg m^−^^3^
Monoclinic, *C*2/*c*	Mo *K*α radiation, λ = 0.71073 Å
Hall symbol: -C 2yc	Cell parameters from 1580 reflections
*a* = 17.0972 (5) Å	θ = 2.5–26.3°
*b* = 9.3528 (3) Å	µ = 7.00 mm^−^^1^
*c* = 11.5334 (4) Å	*T* = 296 K
β = 126.051 (1)°	Block, colourless
*V* = 1491.08 (8) Å^3^	0.20 × 0.18 × 0.16 mm
*Z* = 4	

### Data collection

**Table:**

Bruker SMART APEXII CCD diffractometer	1457 independent reflections
Radiation source: fine-focus sealed tube	1371 reflections with *I* > 2σ(*I*)
graphite	*R*_int_ = 0.019
φ and ω scans	θ_max_ = 26.0°, θ_min_ = 2.6°
Absorption correction: multi-scan (*SADABS*; Bruker, 2005)	*h* = −21→21
*T*_min_ = 0.335, *T*_max_ = 0.401	*k* = −11→11
9665 measured reflections	*l* = −14→14

### Refinement

**Table:**

Refinement on *F*^2^	Primary atom site location: structure-invariant direct methods
Least-squares matrix: full	Secondary atom site location: difference Fourier map
*R*[*F*^2^ > 2σ(*F*^2^)] = 0.015	Hydrogen site location: inferred from neighbouring sites
*wR*(*F*^2^) = 0.039	H-atom parameters constrained
*S* = 1.08	*w* = 1/[σ^2^(*F*_o_^2^) + (0.0184*P*)^2^ + 1.2604*P*] where *P* = (*F*_o_^2^ + 2*F*_c_^2^)/3
1457 reflections	(Δ/σ)_max_ < 0.001
97 parameters	Δρ_max_ = 0.27 e Å^−^^3^
0 restraints	Δρ_min_ = −0.29 e Å^−^^3^

### Special details

**Table:**

Geometry. All e.s.d.'s (except the e.s.d. in the dihedral angle between two l.s. planes) are estimated using the full covariance matrix. The cell e.s.d.'s are taken into account individually in the estimation of e.s.d.'s in distances, angles and torsion angles; correlations between e.s.d.'s in cell parameters are only used when they are defined by crystal symmetry. An approximate (isotropic) treatment of cell e.s.d.'s is used for estimating e.s.d.'s involving l.s. planes.
Refinement. Refinement of *F*^2^ against ALL reflections. The weighted *R*-factor *wR* and goodness of fit *S* are based on *F*^2^, conventional *R*-factors *R* are based on *F*, with *F* set to zero for negative *F*^2^. The threshold expression of *F*^2^ > σ(*F*^2^) is used only for calculating *R*-factors(gt) *etc*. and is not relevant to the choice of reflections for refinement. *R*-factors based on *F*^2^ are statistically about twice as large as those based on *F*, and *R*- factors based on ALL data will be even larger.

### Fractional atomic coordinates and isotropic or equivalent isotropic displacement parameters (Å^2^)

**Table:**

	*x*	*y*	*z*	*U*_iso_*/*U*_eq_	
Br1	0.380531 (15)	0.11870 (2)	0.03979 (2)	0.04022 (8)	
Zn1	0.5000	0.25499 (3)	0.2500	0.02736 (8)	
N1	0.5000	0.4802 (2)	0.2500	0.0253 (4)	
N2	0.58982 (11)	0.31649 (16)	0.18054 (15)	0.0288 (3)	
C1	0.63375 (14)	0.2248 (2)	0.1474 (2)	0.0352 (4)	
H1	0.6256	0.1272	0.1525	0.042*	
C2	0.69093 (14)	0.2696 (2)	0.1056 (2)	0.0392 (4)	
H2	0.7210	0.2033	0.0838	0.047*	
C3	0.70254 (14)	0.4137 (2)	0.0970 (2)	0.0394 (4)	
H3	0.7411	0.4461	0.0700	0.047*	
C4	0.65606 (13)	0.5102 (2)	0.12890 (18)	0.0346 (4)	
H4	0.6620	0.6081	0.1219	0.042*	
C5	0.60056 (12)	0.45792 (18)	0.17152 (16)	0.0268 (4)	
C6	0.54892 (12)	0.55136 (18)	0.21013 (16)	0.0263 (3)	
C7	0.54910 (13)	0.69976 (19)	0.20701 (19)	0.0337 (4)	
H7	0.5815	0.7485	0.1767	0.040*	
C8	0.5000	0.7736 (3)	0.2500	0.0367 (6)	
H8	0.5000	0.8730	0.2500	0.044*	

### Atomic displacement parameters (Å^2^)

**Table:**

	*U*^11^	*U*^22^	*U*^33^	*U*^12^	*U*^13^	*U*^23^
Br1	0.04910 (14)	0.03300 (12)	0.03959 (12)	−0.01116 (8)	0.02667 (10)	−0.00711 (7)
Zn1	0.03545 (17)	0.01984 (14)	0.03395 (16)	0.000	0.02441 (14)	0.000
N1	0.0293 (10)	0.0231 (10)	0.0253 (9)	0.000	0.0170 (9)	0.000
N2	0.0322 (8)	0.0264 (8)	0.0335 (7)	−0.0007 (6)	0.0224 (7)	0.0004 (6)
C1	0.0407 (11)	0.0311 (10)	0.0417 (10)	0.0025 (8)	0.0285 (9)	−0.0011 (8)
C2	0.0372 (11)	0.0487 (12)	0.0398 (10)	0.0025 (9)	0.0271 (9)	−0.0038 (9)
C3	0.0354 (10)	0.0545 (12)	0.0380 (10)	−0.0069 (9)	0.0271 (9)	−0.0022 (9)
C4	0.0367 (10)	0.0359 (10)	0.0339 (9)	−0.0072 (8)	0.0222 (8)	0.0005 (8)
C5	0.0268 (9)	0.0287 (9)	0.0235 (7)	−0.0027 (7)	0.0139 (7)	0.0004 (7)
C6	0.0274 (9)	0.0247 (8)	0.0237 (7)	−0.0027 (7)	0.0133 (7)	0.0007 (6)
C7	0.0360 (10)	0.0268 (9)	0.0351 (9)	−0.0042 (8)	0.0193 (8)	0.0030 (7)
C8	0.0426 (16)	0.0201 (12)	0.0409 (14)	0.000	0.0210 (13)	0.000

### Geometric parameters (Å, °)

**Table:**

Br1—Zn1	2.4179 (2)	C2—H2	0.9300
Zn1—N1	2.106 (2)	C3—C4	1.388 (3)
Zn1—N2^i^	2.1861 (14)	C3—H3	0.9300
Zn1—N2	2.1861 (14)	C4—C5	1.389 (2)
Zn1—Br1^i^	2.4179 (2)	C4—H4	0.9300
N1—C6^i^	1.3441 (19)	C5—C6	1.485 (2)
N1—C6	1.3441 (19)	C6—C7	1.388 (3)
N2—C1	1.336 (2)	C7—C8	1.385 (2)
N2—C5	1.348 (2)	C7—H7	0.9300
C1—C2	1.385 (3)	C8—C7^i^	1.385 (2)
C1—H1	0.9300	C8—H8	0.9300
C2—C3	1.374 (3)		
			
N1—Zn1—N2^i^	74.75 (4)	C3—C2—H2	120.5
N1—Zn1—N2	74.75 (4)	C1—C2—H2	120.5
N2^i^—Zn1—N2	149.49 (8)	C2—C3—C4	119.27 (17)
N1—Zn1—Br1	121.815 (7)	C2—C3—H3	120.4
N2^i^—Zn1—Br1	98.34 (4)	C4—C3—H3	120.4
N2—Zn1—Br1	97.60 (4)	C3—C4—C5	118.78 (18)
N1—Zn1—Br1^i^	121.815 (7)	C3—C4—H4	120.6
N2^i^—Zn1—Br1^i^	97.60 (4)	C5—C4—H4	120.6
N2—Zn1—Br1^i^	98.34 (4)	N2—C5—C4	121.69 (16)
Br1—Zn1—Br1^i^	116.370 (14)	N2—C5—C6	114.99 (14)
C6^i^—N1—C6	120.6 (2)	C4—C5—C6	123.32 (16)
C6^i^—N1—Zn1	119.68 (10)	N1—C6—C7	121.01 (16)
C6—N1—Zn1	119.68 (10)	N1—C6—C5	114.25 (15)
C1—N2—C5	118.88 (15)	C7—C6—C5	124.74 (15)
C1—N2—Zn1	124.80 (12)	C8—C7—C6	118.57 (17)
C5—N2—Zn1	116.32 (11)	C8—C7—H7	120.7
N2—C1—C2	122.43 (18)	C6—C7—H7	120.7
N2—C1—H1	118.8	C7^i^—C8—C7	120.2 (2)
C2—C1—H1	118.8	C7^i^—C8—H8	119.9
C3—C2—C1	118.93 (18)	C7—C8—H8	119.9
			
N2^i^—Zn1—N1—C6^i^	0.68 (9)	C1—C2—C3—C4	−0.6 (3)
N2—Zn1—N1—C6^i^	−179.32 (9)	C2—C3—C4—C5	1.3 (3)
Br1—Zn1—N1—C6^i^	91.12 (8)	C1—N2—C5—C4	−0.1 (2)
Br1^i^—Zn1—N1—C6^i^	−88.88 (8)	Zn1—N2—C5—C4	179.88 (12)
N2^i^—Zn1—N1—C6	−179.32 (9)	C1—N2—C5—C6	179.87 (15)
N2—Zn1—N1—C6	0.68 (9)	Zn1—N2—C5—C6	−0.19 (18)
Br1—Zn1—N1—C6	−88.88 (8)	C3—C4—C5—N2	−0.9 (3)
Br1^i^—Zn1—N1—C6	91.12 (8)	C3—C4—C5—C6	179.12 (16)
N1—Zn1—N2—C1	179.71 (15)	C6^i^—N1—C6—C7	−0.96 (12)
N2^i^—Zn1—N2—C1	179.71 (15)	Zn1—N1—C6—C7	179.04 (12)
Br1—Zn1—N2—C1	−59.30 (14)	C6^i^—N1—C6—C5	179.02 (15)
Br1^i^—Zn1—N2—C1	58.90 (14)	Zn1—N1—C6—C5	−0.98 (15)
N1—Zn1—N2—C5	−0.23 (11)	N2—C5—C6—N1	0.74 (19)
N2^i^—Zn1—N2—C5	−0.23 (11)	C4—C5—C6—N1	−179.33 (14)
Br1—Zn1—N2—C5	120.76 (11)	N2—C5—C6—C7	−179.29 (16)
Br1^i^—Zn1—N2—C5	−121.05 (11)	C4—C5—C6—C7	0.6 (3)
C5—N2—C1—C2	0.8 (3)	N1—C6—C7—C8	1.9 (2)
Zn1—N2—C1—C2	−179.16 (14)	C5—C6—C7—C8	−178.10 (13)
N2—C1—C2—C3	−0.4 (3)	C6—C7—C8—C7^i^	−0.91 (11)

Symmetry codes: (i) −*x*+1, *y*, −*z*+1/2.
